# Mechanical Properties and Durability Performance of Recycled Aggregate Concrete Containing Crumb Rubber

**DOI:** 10.3390/ma15051776

**Published:** 2022-02-26

**Authors:** Robert B. Ataria, Yong C. Wang

**Affiliations:** School of Mechanical, Aerospace and Civil Engineering, University of Manchester, Manchester M1 7JR, UK; yong.wang@manchester.ac.uk

**Keywords:** recycled aggregates, crumb rubber, recycled concrete, compressive strength, tensile strength, durability

## Abstract

Despite extensive research studies, recycled aggregates and worn-out tyres of motor vehicles are still not fully reused and are hence disposed of in ways that are damaging to the environment. Several studies have been carried out on recycled aggregate and rubberized concrete, but very limited studies are conducted on rubber recycled aggregate concrete. This study focuses on the workability, mechanical properties and durability performance of concrete made with 100% recycled aggregates and crumb rubber at different replacement level (5%, 10%, 15% and 20%). The first stage of the study covers the effect of incorporating crumb rubber at different concentration on the workability and mechanical properties of recycled aggregate concrete. The results revealed that the workability and mechanical properties of the recycled aggregate concrete can be used for structural applications when 5% of crumb rubber are used to replace recycled aggregates. The 28-days compressive strength of the rubberized recycled aggregate concrete with 5% crumb rubber concentration is reduced by 21.1% and 32.8% when compared to recycled aggregate concrete and control concrete, respectively. The second stage of the study assesses the durability performance of the recycled aggregate concrete with 5% crumb rubber concentration. The 5% crumb rubber content for durability tests was considered because the ultrasonic pulse velocity tests revealed that the quality of the recycled aggregate concrete is questionable if the concentration of crumb rubber particles is beyond 5%. The durability performance using the surface resistivity test also shows that the chloride ion penetration of recycled aggregates concrete with 5% crumb rubber replacement is moderate using air dried curing technique and high using the water bath curing method. Hence the study suggests the use of rubber recycled aggregate concrete for applications were the exposure condition is not extreme.

## 1. Introduction

Waste tyre disposal is currently causing serious environmental issues all over the world. Every year, approximately I billion waste tyres are generated globally, with 1.6 billion new tyres being produced [1]. Likewise, the amount of construction and demolition (C&D) wastes generated as a result of the increasing demolition of existing infrastructures is a thing of concern. It also is estimated that the UK generated 67.8 million tonnes of non-hazardous C&D waste, of which 62.6 million tonnes (92.3%) was recovered [2]. Studies have tried to utilize recycled aggregates and crumb rubber particles in making new concrete for structural applications [3,4,5], however a limited number of the studies combine both recycled materials to produce concrete [6,7,8]. To tackle the twin challenges of improving properties of recycled aggregate concrete, with or without crumb rubber, and replacing concrete using natural aggregates with recycled aggregate, it is necessary to understand the properties of recycled aggregate concrete with and without crumb rubber for structural applications.

The performance of recycled aggregate concrete generally decreases [9,10,11,12] and the reduction depends on many factors such as the quality of recycled aggregates [13], replacement level in concrete [9,12], water cement (w/c) ratio [14,15], etc. The strength reduction was also attributed to the amount of attached cement matrix on the recycled aggregates [10,12,16], which causes a weak interfacial bond between the attached old cement matrix and the surrounding concrete matrix. Different methods have been devised by authors to enhance the performance of the recycled concrete. Some of these techniques include the addition of extra amounts of cement, use of super plasticizers, incorporation of fly ash, silica fume [9,16,17,18] and the two stage mixing approach [19].

The research results so far also indicate that incorporating crumb rubber in concrete decreases the resulting concretes’ compressive and tensile strengths. This is attributed to the lower strength of rubber particles, and their weak bonding with cement paste [20,21,22,23,24]. However, some researchers have demonstrated that if a small amount of crumb rubber (not more than 5% in volume according to [21,25] and not more than 3% according to [26]) is used to replace mineral aggregates in concrete, then the rubberized concrete could maintain the same mechanical properties as concrete without crumb rubber. It is also possible to enhance the mechanical properties of rubberized concrete as suggested in [27,28,29] by using silica fume. The ultrafine silica fume is believed to create a good bonding between the rubber particles and the surrounding cement paste matrix. Pre-treatment of the rubber particles by soaking in sodium hydroxide NaOH solution before incorporating them into concrete is another method of enhancing the mechanical properties of rubberised concrete, as demonstrated by [6,30]. This was attributed to the benefit of the NaOH solution dislodging the zinc stearate on the rubber surface thereby enhancing the bonding between the rubber powder and the concrete substrate. It was also reported by [23,25,31,32] that rubber particles with small sizes gave higher strength than coarse rubber particles. This was attributed to the formation of larger air voids in the concrete when coarse crumb rubber particles were used [31]. Reference [32] investigated the durability properties of rubberized concrete with up to 30% rubber content. The carbonation depth of rubberized concrete was also greater than that of conventional concrete, and it increased with the rubber content, indicating greater corrosion susceptibility. The study’s findings suggest that rubberized concrete with a rubber content of up to 15% can be used for structural components with sufficient strength and service life. Rubberized concrete is frequently used in low-value applications such as road barriers, concrete paving blocks and playground concrete works. However, using rubberized concrete in structural members is an effective way to improve ductility, which is critical for structural members, particularly in seismic areas. When compared to conventional concrete, the use of crumb rubber in precast concrete panels is also beneficial in terms of sound absorption [33].

The current study assesses the performance of concrete made with recycled aggregate and crumb rubbers at different replacement levels. Different studies have been conducted on concrete performance either made with recycled aggregates or crumb rubber, but very limited studies utilize both recycled materials to make concrete [6,7,8]. Furthermore, there is a lack of information on the durability performance of rubber recycled aggregate concrete; thus, this study aims to fill these knowledge gaps.

## 2. Materials

### 2.1. Material Used

CEM 11/B-V 32.5N Portland fly ash cement complying with [34] was used for this study. Uncrushed natural aggregates of 10 mm size and recycled aggregates with composition shown in Table 1 were used in this work. The recycled aggregates used for this work sourced from Offerton Sand and Gravel (Manchester, UK). The recycled aggregates contain other impurities as detailed in Table 1. According to [35], the classification of recycled aggregates used in this study are low quality RC_80_ (recycled aggregates obtained from concrete products with 20% impurities). The water absorption rates and the densities of both the recycled and natural aggregates are shown in Table 2.

**Figure 1:**
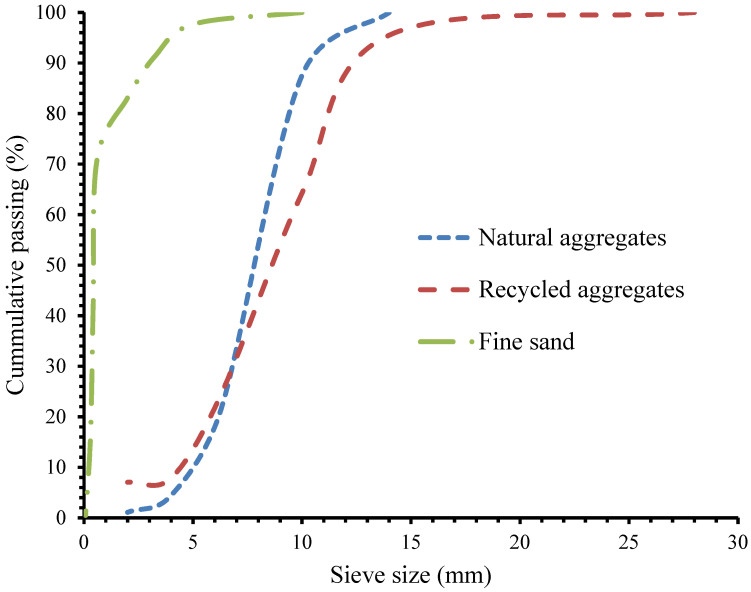
Grading of natural aggregates, recycled aggregates and fine sand [36].

**Figure 2:**
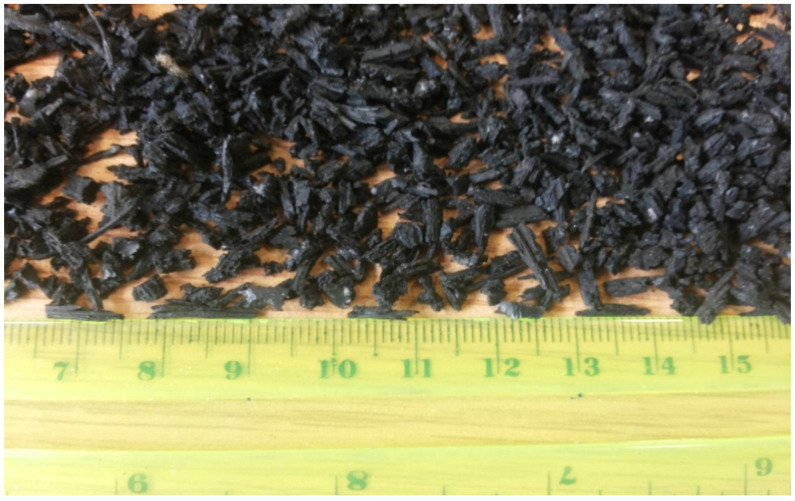
Crumb rubber (8 mm length) particles from worn out tyres.

The grading of the natural aggregates, recycled aggregates are shown in Figure 1. Crumb rubber of 8 mm length and 2mm thickness with an aspect ratio of 4 from worn out vehicle tyres as shown in Figure 2 were supplied by SRC Products Ltd. (Stockport, UK).

### 2.2. Concrete Mix and Specimen Preparation

This study designed a reference concrete of 40 MPa cylindrical compressive strength as shown in Table 3. Crumb rubber was used to replace the coarse aggregates in different concentrations (5%, 10%, 15% and 20%). The experimental programme in Table 4 allows for the systematic investigation and comparison of the effects of different proportions of crumb rubber in recycled aggregate concrete to those of natural aggregate concrete (NAC). The percentages of replacement of recycled aggregates by crumb rubber were carefully selected based on the recommendations made by [22,37,38] for the rubberised recycled aggregate concrete to achieve a substantial proportion of the mechanical properties of the reference concrete. In order to achieve a workable concrete, superplasticizer (1% of cement weight) was added to the recycled aggregate concrete with and without crumb rubber.

The recycled aggregates and fine sand were incorporated into the concrete mixer and mixed for 60 s followed by crumb rubber and 50% of water for another 60 s. Cement was then added for another 30 s and finally the remaining quantity of water was added in 120 s to attain a uniform concrete mix.

### 2.3. Concrete Resistivity of Specimens

For regular reinforced concrete structures using recycled aggregate concrete, durability is a particular concern [9,11,39] because recycled aggregates in their natural crushed state have higher porosity and permeability than natural aggregates and are therefore more susceptible to corrosion of the reinforcement.

In this study, the concentration of crumb rubber particles in recycled aggregate concrete is limited to 5% in order to preserve its mechanical properties. Hence, the durability assessment of the recycled aggregate concrete with 5% of crumb rubber concentration will be conducted.

**Figure 3:**
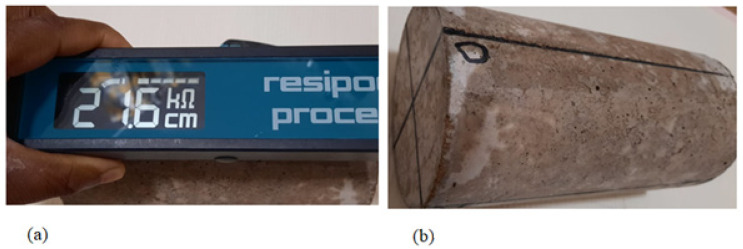
Surface resistivity measurements of cylindrical concrete samples. (**a**) Resistivity measurement on cylinders. (**b**) Sample markings.

The surface resistivity test was used in this study to measure the durability performance (chloride ion penetration) of the recycled aggregate concrete with crumb rubber. The resistivity tests were conducted on cylindrical specimen of size ϕ100 × 200 mm based on the specifications presented in [40]. The four probes are placed on the surface of the cylindrical specimen to produce electrical contact as shown in Figure 3. The two external probes generate pulse of alternating current through the concrete sample and the inner probes measure the electrical potential created. The surface resistivity measurements were taken with a fixed probe spacing of 38 mm with an alternating current frequency of 13 Hz [41,42].

## 3. Experimental Results

### 3.1. Fresh Properties

**Figure 4:**
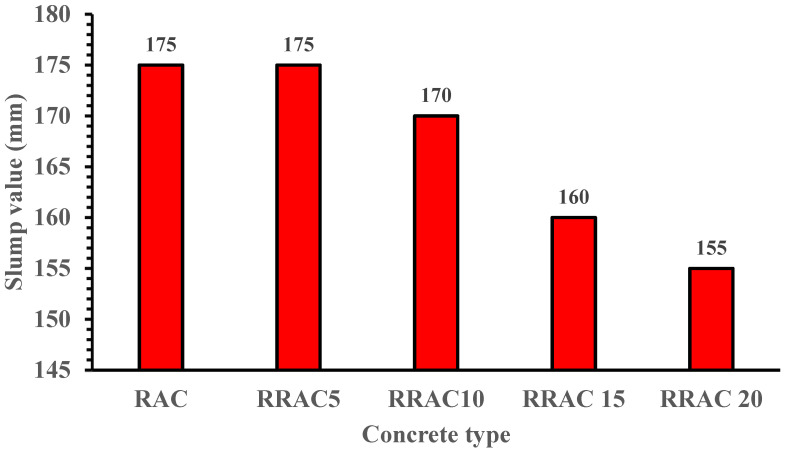
Influence of crumb rubber concentration on the workability of recycled aggregate concrete.

For each mix, slump tests were carried out and results presented to ascertain the workability of the concrete. The workability of all mixes was assessed by means of slump test according to [43]. The concrete was placed in a cone of 300 mm in three layers. At each layer, the concrete is compacted with 25 strokes of a tamping rod. After filling and compacting the top layer, the surface of the concrete is struck off by means of sawing and the rolling action of the compacting rod. The mould is then carefully lifted within a time interval of 2 to 5 s. Figure 4 shows the effect of the crumb rubber and recycled aggregates on the workability of concrete.

The workability of the recycled aggregate concrete was enhanced to level comparable to the reference concrete (165 mm) by adding superplasticizer (1% of the cement weight). The added superplasticizer compensates for the water absorbed by the recycled aggregates during mixing process [18,44,45]. However, adding crumb rubber reduces the workability of the recycled aggregate concrete. This can be attributed to the crumb rubber surface texture and water absorbability of the crumb rubber particles. The observed results are similar to those reported by [23,42,46].There is no significant difference in workability when 5% of the recycled aggregates were replaced with crumb rubber particles in recycled aggregate concrete. This is due to the crumb rubber’s slightly higher water absorption than the replaced recycled aggregates. According to [47], the water absorption of crumb rubber less than 50mm is 6.7%, while recycled aggregates, as shown in Table 2, have a water absorption of 5.77%. Hence, 5% crumb rubber replacement level did not have influence on workability. However, a sharp reduction in workability was observed for rubber recycled aggregate concrete when more than 5% crumb rubber particles were incorporated into the recycled aggregate concrete.

### 3.2. Hardened Properties

#### 3.2.1. Compressive Strength

**Figure 5:**
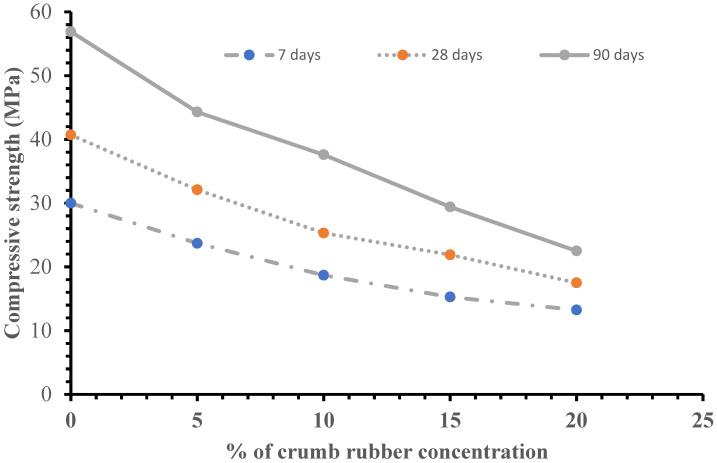
Influence of crumb rubber content on the compressive strength of recycled aggregate concrete.

For each mix, three concrete cubes were tested as per specifications made in [48] and the results presented are the average values for 7, 28 and 90 days of curing. As expected, the results in Table 5 and Figure 5 reveals that addition of crumb rubber particles in the recycled aggregate concrete reduces its compressive strength. The reduction of the compressive strength was attributed to the low elastic modulus and high poisons ratio of the crumb rubber capable of initiating premature cracking under loading condition [23,49,50]. Weak bonding between the rubber particles and cement paste could also lead to reduction in compressive strength [51]. However, the recycled aggregate concrete maintains most of its properties when the crumb rubber concentration is limited to 5%.

The results of the ultrasonic pulse velocity in Table 5 were used to ascertain the quality of already cast concrete. The ultrasonic pulse velocity tests were based on the specifications made in [52]. The pulse velocity of the recycled aggregate concrete drop by 6.9% compared to reference concrete mainly due to the micro cracks sustained by the recycled aggregates during extraction. Addition of crumb rubber to the recycled aggregate concrete further drop the pulse velocity in Table 5 and is attributed to the entrapped air by the crumb rubber particles, as well as the low ultrasonic wave velocity in crumb rubber. However, based on the criteria made in Table 6, the quality of the rubber recycled aggregate concrete in Table 5 is questionable if the rubber content is more than 5%.

**Figure 6:**
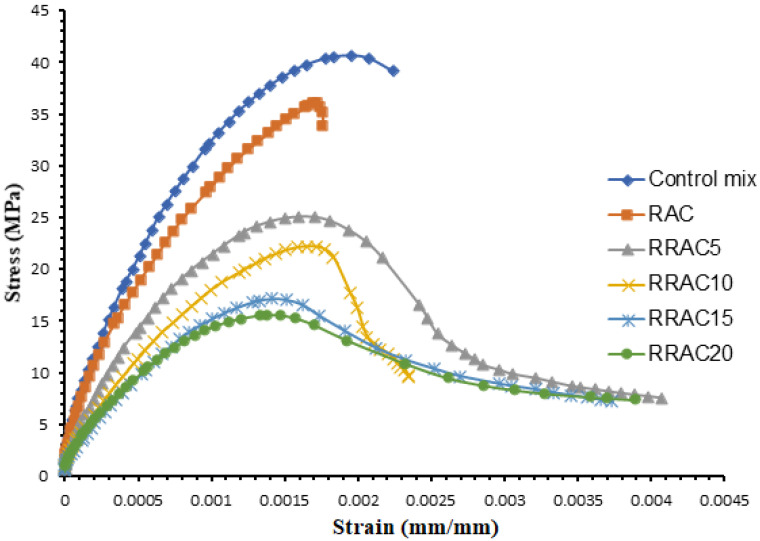
Compressive stress strain relationships of recycled aggregate concrete with different amounts of rubber particles.

Figure 6 shows the compressive strength of the concrete mixes subject to axial monolithic loading. The stress strain results in Figure 6 shows higher ductility of the concrete with crumb rubber particles compared to the conventional concrete. 

The failure mode of the concrete mixes without crumb rubber is brittle. However, addition of crumb rubber enhances the ductility of the recycled concrete mixes. This due to the higher deformability of the crumb rubber particles used in the replacing the recycled aggregates.

#### 3.2.2. Tensile Strength

**Figure 7:**
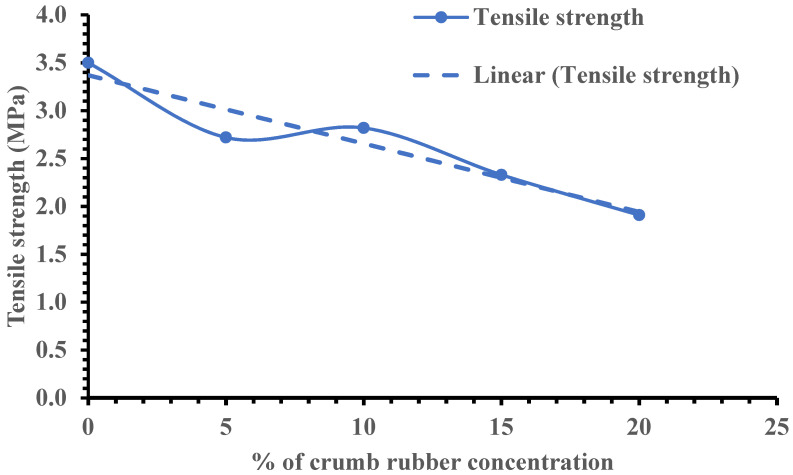
Influence of crumb rubber content on the tensile strength of recycled aggregate concrete.

Three split tensile strength tests were carried out for each mix based on the specifications in [53] and the average results are presented in Figure 7, which shows how the amount of crumb rubber influenced the splitting tensile strength of rubber recycled aggregate concrete. A linear reduction is the best fit, ranging from 14.3% at 5% crumb rubber to reductions of 21.4%, 35.7% and 45.4% at 10%, 15% and 20% of crumb rubber, respectively, for rubber recycled aggregate concretes.

### 3.3. Durability of the Recycled Aggregate Concrete with Crumb Rubber

The samples for the concrete resistivity tests were cured for 28 days prior to testing. Two curing techniques were adopted in this study; some samples are fully submerged in tap water at a temperature of (20 ± 2) °C and relative humidity ≥95% while others are wrapped with damp cloth at a temperature of (20 ± 5) °C. The samples cured in tap water were tested under surface saturation condition. All the samples are marked at four locations equally spaced at 90 degrees as shown in Figure 3 prior to curing process. The surface resistivity tests for surface saturated samples and air-dried samples were carried out after 28 days curing. Excess water on the samples surface cured in water were wiped off with damped cloth. The probes of the resistivity equipment are dipped in water prior to tests for air dried samples to ensure proper surface contact. Resistivity measurements of all samples were repeatedly taken at four different locations (total of eight readings on each sample) to ensure quality control applications as specified in [40].

**Figure 8:**
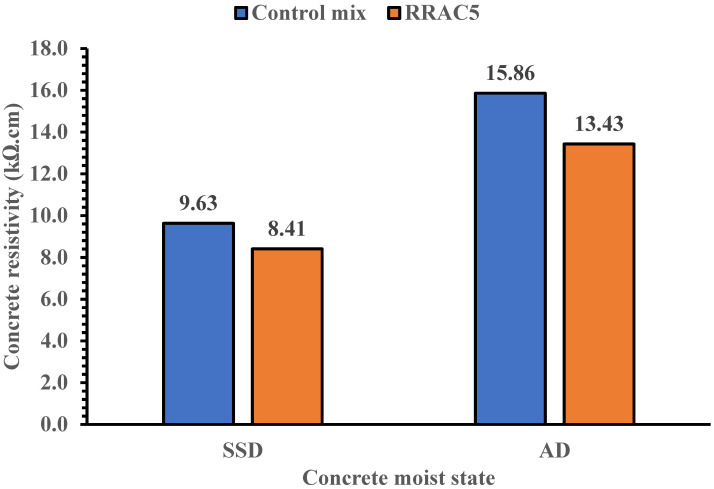
Surface resistivity of concrete mixes at saturated surface dried and air-dried state for 7 days curing.

**Figure 9:**
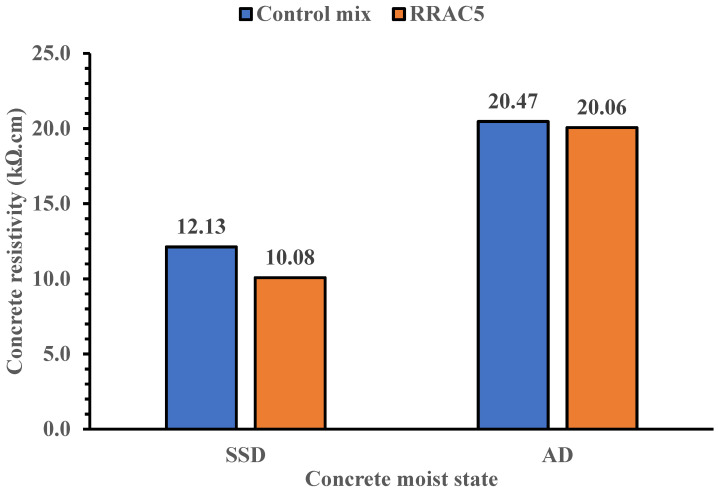
Surface resistivity of concrete mixes at saturated surface dried and air-dried state for 28 days curing.

The surface resistivity of concrete mixes cured for 7 and 28 days are shown in Figure 8 and Figure 9. The chloride ion penetration of concrete mixes for rubber recycled aggregate concrete under saturated surface dried state is high based on the specifications made in Table 7. This may be attributed to the voids in recycled concrete due to the presence of crumb rubber particles, hence creating a path for ingress of fluids into the concrete. This can also be illustrated with the ultrasonic pulse velocity tests in Table 5. The ultrasonic pulse velocity of the recycled aggregate concrete reduces with the increase in crumb rubber concentration, indicating the presence of voids in the concrete.

However, under air dried state the durability performance of the recycled concrete with 5% crumb rubber concentration is moderate based on the Table 7 specifications. The result of the recycled concrete is also comparable to the control mix under the air-dried curing method. Hence, the use of recycled concrete containing rubber content of limited amount (5%) could be useful for construction applications, especially where exposure condition is not extreme or critical. 

## 4. Conclusions

The effect of crumb rubber on the strength and penetration of chloride ion in recycled aggregate concrete was investigated using the surface resistivity test method. Different concentrations (5%,10%, 15% and 20%) of crumb rubber in recycled aggregate concrete were considered in the study. The results clearly shown that addition of crumb rubber reduces the compressive strength of the recycled aggregate concrete moderately when its concentration is limited to 5%. The study also observes the resistivity of chloride ions for recycled aggregate concrete with 5% crumb rubber concentration. Based on the ultrasonic pulse velocity results, the use of recycled aggregate concrete with crumb rubber content beyond 5% is questionable.

The results of the surface resistivity tests shows that the resistivity of the recycled aggregate concrete to chloride ion penetration is moderate under the air dried curing technique. However, using the water bath technique, the resistivity was found to be low based on the specifications listed in Table 7. Hence, the study suggests the application of recycled aggregate concrete with crumb rubber in the construction industry where the exposure conditions are not extreme. The rubber recycled aggregate concrete could be useful, for example, in making concrete wall panels, concrete lintels and road kerbs where the criteria for exposure conditions for durability are not so critical.

Furthermore, the ductility of recycled concrete with crumb rubber is found to be greater than that of recycled concrete without crumb rubber. As a result, using rubberized concrete for structural members subject to seismic loads is a promising potential application. Rubberized recycled concrete is an alternative solution for meeting sustainability targets and reducing embodied carbon in the construction industry. However, its limitations are the reduction in strength and the willingness of contractors to use the concrete product on new projects due to the previously mentioned associated drawbacks.

### Further Research

Further research on the durability performance of the recycled concrete with crumb rubber concentration using different mix ratios is needed. Pre-treatment of the crumb rubber particles could also be considered when assessing the durability of the recycled concrete.

## Figures and Tables

**Table 1:** Composition of natural and recycled aggregates.

Composition	Proportion
Natural aggregates	
Quartzite	79%
Sandstone	6%
Basalt	5%
Others	10%
Recycled Aggregates	
Recycled Aggregates	81%
Bricks	13%
Dust	6%

**Table 2:** Water absorption rates and densities of natural and recycled aggregates.

Type	Apparent Particle Density	Particle Density on Oven Dry Bases	Particle Density on Saturated and Oven Dry Bases	Water Absorption (%)
Natural aggregate	2.69	2.62	2.65	1.05
Recycled aggregates (RA)	2.61	2.27	2.40	5.77

**Table 3:** Concrete mix composition of the reference concrete.

Mix Type	Cement (kg)	Water (kg)	w/c	Sand (kg)	Coarse Aggregates (kg)
NAC	550	220	0.4	626	939

**Table 4:** Experimental programme.

Specimen	Natural Aggregates	Recycled Aggregates (RA)	Crumb Rubber	Sand
Designation	(% by Weight)	(% by Weight)	(% by RA Weight)	(% by Weight)
NAC	100	-	-	100
RAC	-	100	-	100
RRAC5	-	100	5	100
RRAC10	-	100	10	100
RRAC 15	-	100	15	100
RRAC 20	-	100	20	100

NAC- Natural aggregate concrete; RAC- Recycled aggregate concrete; RA- Recycled aggregate; RRAC5, RRAC10, RRAC15 and RRAC20- Rubber recycled aggregate concrete with 5, 10, 15 and 20 percent of crumb rubber content respectively of recycled aggregate weight.

**Table 5:** Experimental results of the concrete hardened properties.

Specimen	Compressive Strength (MPa)			Tensile	Ultrasonic Pulse
Type	7 days	28 days	90 days	Strength MPa	Velocity (KM/s)
NAC	34.6	47.8	60.4	4.11	4.19
RAC	30	40.7	56.9	3.50	3.90
RRAC5	23.7	32.1	44.3	2.72	3.72
RRAC10	18.69	25.3	37.6	2.82	3.61
RRAC 15	15.28	21.9	29.4	2.33	3.54
RRAC 20	13.24	17.5	22.5	1.91	3.41

**Table 6:** Concrete classification based on Ultrasonic pulse velocity [52].

Ultrasonic Pulse Velocity (m/s)	Concrete Classification
V > 4575	Excellent
4575 > V > 3660	Good
3660 > V > 3050	Questionable
3050 > V > 2135	Poor
V < 2135	Very poor

**Table 7:** Comparison of chloride penetrability levels established for standards based on electrical resistivity (AASHTO TP 95) and charged passed (ASTM C1202) [40].

Chloride Ion Penetrability	AASHTO TP 95, kΩ.cm	ASTM C1202, Coulombs
High	<12	>4000
Moderate	12 to 21	2000 to 4000
Low	21 to 37	1000 to 2000
Very Low	37 to 254	100 to 1000
Negligible	>254	<100
